# Plant-Based Biostimulant as Sustainable Alternative to Synthetic Growth Regulators in Two Sweet Cherry Cultivars

**DOI:** 10.3390/plants10040619

**Published:** 2021-03-24

**Authors:** Boris Basile, Natalie Brown, José Miguel Valdes, Mariateresa Cardarelli, Pasquale Scognamiglio, Alessandro Mataffo, Youssef Rouphael, Paolo Bonini, Giuseppe Colla

**Affiliations:** 1 Department of Agricultural Sciences, University of Naples Federico II, 80055 Portici, Italy; pasquale.scognamiglio2@unina.it (P.S.); alessandro.mataffo@unina.it (A.M.); youssef.rouphael@unina.it (Y.R.); 2 Research and Development Department-In-Pacta, Santiago, Chile; nbrown@in-pacta.cl (N.B.); jmvaldes@in-pacta.cl (J.M.V.); 3 Consiglio Per la Ricerca in Agricoltura e L’analisi Dell’economia Agraria, Centro di Ricerca Orticoltura e Florovivaismo, 84098 Pontecagnano Faiano, Italy; mteresa.cardarelli@crea.gov.it; 4 oloBion—OMICS LIFE LAB, Barcelona, Spain; pb@ngalab.com; 5 Department of Agriculture and Forest Sciences, University of Tuscia, 01100 Viterbo, Italy

**Keywords:** *Prunus avium* L., sustainable agriculture, fruit cracking, calcium, skin color

## Abstract

Sweet cherry is a high value crop and the economic success of its cultivation depends not only on yield but also on fruit visual and nutritional quality attributes that influence consumer acceptability, as well as on fruit post-harvest performance and resistance to cracking. During the last few decades, cherry growers have tried to achieve these goals through exogenous applications of synthetic plant hormones and/or nutrients, but there is growing concern about the sustainability of the extensive use of these compounds in agriculture. For this reason, there is increasing interest in the possible adoption of different classes of biostimulants as sustainable alternatives to plant growth regulators. This research aimed to study the impact of foliar application of a novel tropical-plant extract, performed between full bloom and fruit set, on the yield and fruit quality of two important commercial sweet cherry cultivars, Kordia and Regina. The experimental design included a commercial control involving the application of a cytokinin promoter. In both cultivars, the tropical-plant extract induced significant increases in fruit yield. In addition, in the cultivar Kordia, the tropical-plant extract enhanced fruit calcium concentration, soluble solids content, flesh firmness, and skin color by 26.2%, 11.8%, 6.7%, and 12.0% (of fruits with mahogany skin color), respectively. Our results suggest that the tropical-plant extract tested as a biostimulant may be a sustainable and effective alternative to the exogenous application of synthetic hormones for sweet cherry cultivation.

## 1. Introduction

Global sweet cherry (*Prunus avium* L.) cultivation has increased steadily during the last decade, reaching a total of 2.5 million tons in 2018 [1]. The increased commercial interest for this crop is probably mainly related to the introduction of new size-controlling rootstocks that allowed the adoption of new training systems, the increase in planting densities, and the introduction of over-head protection systems against rain, hail, and birds. These innovations allowed significant decreases in labor costs (for pruning and harvest), an increase in orchard productivity and a decrease in yield losses due to biotic and abiotic stresses. Nowadays, after Turkey and the United States of America, Chile is the third most important sweet cherry producing country in the world (with a cultivated surface of 30,179 ha and a total yield of 155,935 tons in 2018; FAOSTAT, 2020), and it is the top country in terms of production for export (184,566 tons in 2018; [1]).

To better meet the increased demand of the market for sweet cherries, a large body of research has focused (a) on the identification of the most important fruit qualitative traits driving consumers’ liking of cherries [2,3,4,5] and (b) on defining pre- and post-harvest strategies to improve these fruit attributes and maintain them during handling, packaging, storage and transport required for exportation [6,7,8,9]. Among the external fruit qualitative attributes, large fruit size and dark and uniform fruit skin color are the ones mainly positively affecting the consumer’s liking of fruit appearance [3,4]. This is the reason why cherry fruit size is still the main qualitative trait positively affecting cherry value [10]. Moreover, considering the importance of cherry appearance in consumer acceptance, the occurrence of fruit cracking, a severe physiological disorder causing evident cracks of the cherry fruit cuticle, represents a serious threat to fruit yield and quality [11]. Indeed, cracked cherries have no commercial value for fresh market, but they may be used for processing (when they do not present fungus attacks). Other studies have also highlighted that consumers’ liking/acceptance of cherries increases when fruit flesh is firm, even though it should not be “too firm” (>4.71 N) [5,12]. Considering that cherries are perishable fruits, fruit softening represents a major issue in the management of cherry fruit post-harvest, especially when exportation aims at long-distance transport.

Due to the commercial relevance of certain qualitative traits, cherry growers and packing and export companies are keen on adopting strategies suitable to improve exterior quality (large fruit size, dark and uniform skin color, absence of surface defects due to cracking, firm fruit flesh). Research has focused on improving these cherry qualitative traits by using the exogenous application of plant hormones and nutrients [9,13]. Pre-harvest applications of gibberellic acids can stimulate cherry fruit growth [9,14] and increase flesh firmness at harvest [14,15,16], but they were also reported to delay fruit ripening as suggested by the decrease in the fruit skin color intensity, soluble solids content, polyphenol concentration and antioxidant activity of cherries at harvest [15,16]. Conversely, the combined application of gibberellic acid and the steroid hormone homobrassinolide was effective in alleviating the negative effect of gibberellins on the concentrations of anthocyanins and phenolics and on soluble solids content [17]. Cherry fruit flesh firmness was improved by pre-harvest sprayings of calcium alone [13] or in combination with gibberellic acid [18], resulting in a decrease in fruit susceptibility to cracking [18,19]. The application of cytokinins at early stages of fruit development [9] or of synthetic auxins at pit hardening [20] has also been reported to stimulate sweet cherry fruit growth and, thus, its final fruit size. Furthermore, in plants, auxins have also been demonstrated to play an important role in calcium translocation and uptake into developing fruits [21].

Despite the demonstrated effectiveness of synthetic hormones and fertilizers’ application in improving cherry yield and qualitative traits [22,23], their extensive use has become undesired as a consequence of the worldwide increasing consensus among scientists and policy makers that modern agriculture needs to meet new sustainability goals by reducing the adoption of environment-impacting agrochemical inputs [24,25]. In this respect, biostimulants are considered to be environmentally friendly, effective, alternative solutions to enhance plant vegetative growth, fruit yield and quality and to increase plant tolerance to abiotic stresses by modulating several aspects of plant physiology (photosynthesis, hormone metabolism, nutrient uptake and translocation, secondary metabolism, etc.) [26,27,28,29]. Despite the significant amount of research carried out on different fruit tree species [30], scientific evidence supporting the biostimulant activity on sweet cherry is still poorly explored. Vercammen et al. [31] suggested that foliar applications of a commercial product containing seaweed extracts (applied two–four weeks before harvest) induced a decrease in the percentage of cracked fruits of up to around 10%. Recently, foliar applications of *Ascophyllum nodosum* extract and calcium were reported to increase organic acid concentration in the fruits of two sweet cherry cultivars (Skeena and Sweetheart) [7]. To the best of our knowledge, there is no published information about the effects of biostimulant application on the yield components of cherry trees.

Based on these findings and with the aim of improving fruit quality, the adoption of a combined use of calcium and hormones is becoming a common management practice in important commercial cherry tree growing districts [9], such as Chile. Lately, several plant extracts obtained from species such as American or French oak [32,33], grapevines [34,35,36] and tropical plants [37,38] have been reported to exert interesting biostimulant effects in horticultural crops, but they were never tested in sweet cherries. This research was designed to study the impact of a tropical-plant extract on the yield components and fruit quality of two sweet cherry cultivars.

## 2. Results

### 2.1. Fruit Yield, Fruit Fresh Weight, Size and Qualitative Traits

Tropical-plant-extract biostimulant (TPEB) application induced a significant increase in fruit yield in both cultivars (by 7.4 and 13.1% in Kordia and Regina trees, respectively) (Table 1). Crop load was significant higher (7.5%) in Regina trees sprayed with the TPEB compared to the control tree (Table 1), whereas in Kordia trees no difference was found between treatments in this parameter (an average of 1124 fruits/tree).

In both cultivars, fruit diameter at harvest was significantly larger in trees sprayed with the TPEB than in control trees (2.5% and 2.1% in Kordia and Regina trees, respectively) (Table 2). The application of the TPEB induced a decrease in fruit fresh weight (5.9%) in Kordia trees (Table 2). Conversely, in Regina trees the fruit fresh weight was significantly increased (8%) by the application of the TPEB compared to the control (Table 2).

**Figure 1:**
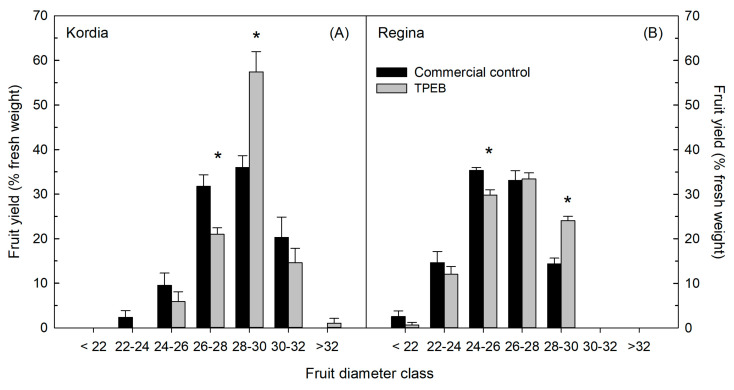
Fruit diameter distribution in “Kordia” (**A**) and “Regina” (**B**) in percentage of the yield, treated with Commercial control management and Tropical-plant extract biostimulant (TPEB). Vertical bars represent the standard error of the mean. Within each panel and separately for each fruit diameter class, asterisks indicate significant differences between treatments according to t-test (*p* ≤ 0.05).

In both cultivars, fruit distribution into commercial fruit diameter classes was significantly affected by the application of the TPEB (Figure 1). Trees sprayed with the TPEB had a significantly higher fraction of fruit yield in the 28–30 mm size class (57.4% and 24.1% in Kordia and Regina trees, respectively) compared to the control (36.0% and 14.3% in Kordia and Regina trees, respectively), whereas in trees sprayed with the TPEB a decrease in the percentage of fruit yield, compared to the control, was found in the size classes 26–28 mm and 24–26 mm in Kordia and Regina trees, respectively.

In Kordia, the application of the TPEB induced an increase in soluble solids content (2.2 °Brix corresponding to 11.8%), flesh firmness (6.7%), and calcium content (26.2%) in fruits at harvest compared to the control (Table 2). Regina trees sprayed with the TPEB had a lower soluble solids content (7.4%) in fruit at harvest compared to control trees (Table 2), whereas for this cultivar, no difference was found between treatments in flesh firmness and calcium content in fruits at harvest (on average 323.6 g/m^2^ and 12.4 mg/100g, respectively).

**Figure 2:**
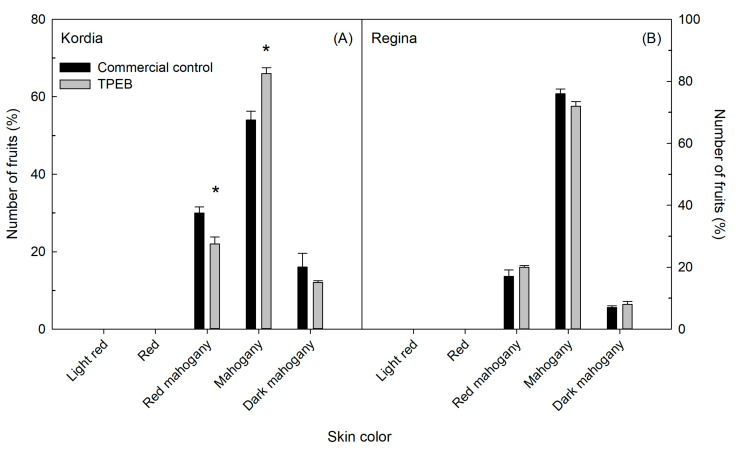
Skin color distribution in “Kordia” (**A**) and “Regina” (**B**) in percentage of the sampled fruits, treated with Commercial control management and Tropical-plant extract biostimulant (TPEB). Each fruit was visually examined and assigned to one of the five skin color classes. Vertical bars represent the standard error of the mean. Within each panel and separately for each fruit skin color class, asterisks indicate significant differences between treatments according to t-test (*p* ≤ 0.05).

The application of the TPEB also increased the percentage of fruit with a mahogany skin color (66% compared to 54% of control trees) and decreased the percentage of fruits with a red mahogany skin color (22% compared to 30% of control trees) (Figure 2A). In Regina trees, no differences between the two treatments were found in fruit distribution in skin color classes (Figure 2B).

### 2.2. Composition of Leaf Petiole Sap

In Kordia trees sprayed with the TPEB, the Ca concentration in petiole sap was three-fold the concentration in control trees (222.7 and 68.2 mg/L, respectively), whereas in the cultivar Regina this parameter was not affected (an average of 11.0 mg/L). In both cultivars, the treatments did not affect the petiole sap concentration of K, Na, NH_4_, NO_3_ and Mg (data not shown).

## 3. Discussion

In both sweet cherry cultivars, the plant extract studied in this research induced a significant increase in fruit yield, but, interestingly, in the two varieties this effect was due to the stimulation of different yield components (Table 1 and Table 2). This suggests that differing biological processes of cherry tree reproductive growth may have been affected depending on the genotype. Indeed, the increment in fruit yield induced by the TPEB was mainly related to an increase in fruit fresh weight in Kordia trees, whereas in the cultivar Regina this effect was related to an increase in both the number of fruits per tree and fruit fresh weight (Table 1 and Table 2). The number of fruits per tree is mainly determined at fruit set, which is a complex biological process (a) inducing the ovary to start growing, (b) determining its transition to fruit, and (c) representing the very first stage of fruit development. In fruit tree cultivation, a successful fruit set is a required condition to reach the desired crop load and fruit yield. Fruit set includes the activation of a complex cascade of metabolic pathways and morphological transformations which are generally triggered by the pollination/fertilization event [39]. This is particularly critical in the commercial cultivation of fruit-tree species such as sweet cherries, where most of the cultivars are self-sterile and there is a need for cross-pollination among inter-compatible varieties. It is well acknowledged that fruit set is regulated mainly by the accumulation of three hormones, auxin, gibberellins (GAs), and cytokinin [40,41]. Furthermore, in the absence of pollination/fertilization, exogenous applications of auxins, cytokinin, or gibberellins were reported to induce parthenocarpy in many cultivated crops [40,41], including sweet cherries [42]. Our results suggest that the application of the TPEB during bloom may exert a stimulating hormone-like effect on the fruit set of cherry trees (often referred to as an auxin-like effect). The biostimulant activity of plant extracts is thought to be mainly related to the presence of mixtures of free amino acids, oligo- and polypeptides that act as signaling molecules triggering important plant molecular and physiological processes [43,44]. Among the major mechanisms involved in their biostimulant activity, plant extracts were reported to (a) boost auxin- and gibberellin-like activities, (b) trigger key enzymes involved in N uptake and C metabolism, and (c) stimulate enzymes involved in secondary metabolism (antioxidant compound and pigment biosynthesis) [45,46,47,48,49,50]. The positive effect of the TPEB on crop load we found in our study is particularly relevant for the self-sterile cultivars Kordia and Regina, which are often classified as “shy bearer” varieties, because of their tendency to have unsatisfactory fruit set in many important cherry growing areas including Chile [51]. However, in our study, the TPEB significantly affected fruit set only in Regina trees, whereas no effect was detected in the cultivar Kordia. The comparison between the crop load of the two cultivars (lower in Kordia than in the Regina trees; Table 1) indicates that other factors may have intervened and interacted. Possible hypotheses may be related to differences between the cultivars in: (a) tree fertility (number of flowers per spur, number of spurs per tree); (b) the specific weather conditions (air temperature, rainfall, etc.) occurring during the phenological stages bloom-fruit set; (c) the sensitivity to the plant-extract. Additional studies are required to better understand these differential responses.

In both cultivars, the tested TPEB stimulated fruit growth resulting in an increased fruit size at harvest (Table 2 and Figure 1). Interestingly, this effect was also significant in Regina trees despite the increase in crop load induced by the TPEB. Indeed, increases in crop load may cause unfavorable source-sink relations resulting in a decrease in fruit growth [52]. Like in other stone fruit species, cherry fruit growth follows a double-sigmoid growth curve, characterized by two intense growth periods (Stages I and III) separated by a phase of slow growth during which pit hardening occurs (Stage II) [53,54,55,56,57]. Fruit growth during Stage I is mainly due to cell division, whereas in Stage II the growth is mainly due to cell enlargement [55,56]. Due to this large biological difference between Stages I and III, fruit growth during these phases is differentially regulated by hormonal signaling. Cytokinins are well known to stimulate cell division, and the exogenous application of these hormones during this stage was reported to increase fruit size at harvest in sweet cherry [9] and other fruit tree species [58]. Conversely, exogenous gibberellins are more efficient in stimulating fruit growth when applied during the cell expansion stage in cherry trees [9] and other fruit tree crops [59]. Considering that in our study the TPEB was applied at very early stages of fruit development (during cell division in the ovary or in the fruitlet), the results obtained suggest that this extract may have induced cytokinin-like effects (Table 2 and Figure 1), as previously reported for other biostimulants [60,61]. Interestingly, this stimulating effect on fruit growth was even stronger than that induced by the cytokinin promoter applied to the trees of the commercial control. In several studies, biostimulants are reported to affect fruit growth as a consequence of an improved plant nutritional status [30]. However, in the case of our study, this does not appear the case since the analyses of the petiole sap composition highlighted that the TPEB increased only Ca concentration in the cultivar Regina (not in Kordia), whereas in both cultivars, K, Na, NH_4_, NO_3_ and Mg concentrations were not affected.

In the cultivar Kordia, the TPEB, in addition to stimulating fruit growth, also positively affected other important fruit cherry qualitative traits such as fruit firmness, soluble solids content, and skin color (Table 2). The increase in fruit firmness was probably related to the higher fruit calcium concentration we found in the TPEB treatment compared to the commercial control (Table 2). It is undisputed that calcium-pectin cross-links play a major role in determining the strength of cell walls and, therefore, the physical and structural properties of fruit [21]. Auxins are thought to be involved in calcium transport and its uptake by the fruit [21]. Thus, our results suggest that the TPEB may exert an auxin-like activity in sweet cherries, resulting in a better calcium nutrition of the sweet cherry fruit. The increase in fruit flesh firmness measured in the TPEB treatment has interesting implications because this parameter positively correlates to consumers’ liking/acceptance of cherries [5,12] and may result in an enhanced suitability of the cherries for post-harvest handling and transport. This is very important for producer countries such as Chile that significantly invest in cherry exportation. In addition to the effect on fruit flesh firmness and its softening rate, an enhanced calcium concentration may result in a lower susceptibility of the cherries to fruit cracking [62]. More specifically designed experiments will be required to test the hypothesis of whether the application of the TPEB can also decrease the occurrence of cherry cracking.

In the cultivar Kordia, the TPEB also appeared to stimulate the accumulation of carbohydrates in the fruits, as suggested by the increase in soluble solids content measured in the cherries at harvest (Table 2). This hypothesis is also consistent with the improvement in fruit skin color we measured in the same treatment. Indeed, anthocyanins are secondary metabolites whose biosynthesis also depends on carbohydrate availability [63,64,65]. Plant extracts and plant-derived protein hydrolysates were previously reported to boost C metabolism and to stimulate the biosynthesis of secondary metabolites such as pigments, phenolic comp, and ascorbic acid [66]. Previous studies on sweet cherries demonstrated that inducing an increase in carbohydrate availability for ripening fruits by fruit flower/fruit thinning can result in an increase in soluble solids and anthocyanin concentration and in darker fruit skins [67,68]. Interestingly, the positive effects of the TPEB on soluble solids content and fruit color were not found in the fruits of the cultivar Regina (Table 2 and Figure 2B). This was also the case for the fruit calcium concentration and flesh firmness at harvest (Table 2). This suggests a lower sensitivity of the cultivar Regina to the application of the TPEB compared to Kordia. Another possible hypothesis to explain this differential response may be due to the different effect the TPEB had on fruit set in the two cultivars (Table 1). Indeed, in Regina the TPEB induced a 7.5% increase in crop load, whereas in Kordia this parameter was unaffected. The increase in crop load may have determined source-sink relations that were less favorable for carbon partitioning to fruits that may have counter-balanced and masked any positive effect of the TPEB on these specific fruit qualitative traits. Previous studies reported that fruit soluble solids content and phenolic acid concentration decreased progressively with increasing crop load [52,69]. The decrease in soluble solids content that we measured in Kordia trees sprayed with the TPEB appears to support this hypothesis (Table 2).

## 4. Materials and Methods

### 4.1. Experimental Site, Plant Material and Experimental Design

The study was conducted in a commercial sweet cherry (*Prunus avium* L.) orchard located in San Fernando, Libertador Bernardo O’Higgins Region, Chile (34°38′59.92″ S, 70°49′12.64″ O; 570 m a.s.l.) during the growing season which started in 2017 and ended in 2018. The experiment was carried out on two cultivars, Kordia and Regina. All trees, planted in 2007, were grafted onto Gisela^®^ 6 rootstocks and trained to a Spanish Bush system. Tree spacing was 4.5 m × 2.5 m (corresponding to a planting density of 889 trees/ha) and row orientation was North–South. From bud break to leaf drop the following total amounts of nutrients were provided to the trees of all treatments through 6 fertigations (mono-ammonium phosphate, potassium nitrate, ammonium nitrate, magnesium nitrate): 283 kg N/ha, 39 kg P/ha, 103 kg K/ha, 58 kg Mg/ha. Two foliar fertilization strategies were compared (Table 3) according to a randomized complete-block design with three replicates. The commercial control treatment included the following four foliar sprays: (a) the first foliar spraying consisted of the application of a commercial *Ascophyllum nodosum* seaweed extract (Stimplex; ANASAC, Providencia, Chile) at a rate of 3.75 L/ha, plus a commercial organic fertilizer (Defender Ca; Kenya Biologics Ltd., Runyenjes, Kenya) containing 14% of calcium complexed with 6% of amino acids at a rate of 4.5 L/ha, applied when 20% of flower buds were at the phenological stage “sepals open” (Stage D, “flower bud open” according to Baggiolini [70]; Stage 57 according to the BBCH scale as described by Fadón et al. [71]); (b) the following three foliar sprays consisted of the application of 4.5 L/ha of Defender Ca and 2 L/ha of a commercial cytokinin promoter (Citogrower; Kenya Biologics Ltd., Kenya) at three phenological stages: “full bloom” (Stage F [70]; BBCH Stage 65 [71]),”full petal fall” (Stage G [70]; BBCH Stage 67 [71]), and 7 days after “full petal fall”.

The biostimulant-based treatment included the following four foliar sprays: (a) the first consisted of the application of a commercial *Ascophyllum nodosum* seaweed extract (Stimplex; ANASAC, Chile) at a rate of 3.75 L/ha plus a commercial organic fertilizer (Myr Calcio; Hello Nature USA Inc., Anderson, IN) containing 5% of calcium (CaO) and 5% of vegetal amino acids at a rate of 3 L/ha, applied when 20% of flower buds were at the phenological stage “sepals open”; (b) the other three sprays consisted of the application of Myr Calcio (3 L/ha) plus a commercial tropical-plant extract (Auxym; Hello Nature USA Inc., Anderson, IN) (1.5 L/ha) at three phenological stages, “full bloom”, ”full petal fall”, and 10 days after “full petal fall”. This latter product was obtained through a water extraction and fermentation of tropical plants. The composition of this was fully described by Colla et al. [38]. Briefly, this biostimulant contains phytohormones (auxins: 1.81 mg/kg; cytokinins: 0.29 mg/kg), amino acids (51.9 g/kg), vitamins (niacin: 3.3 g/kg; vitamin C: 1.0 g/kg; vitamin E: 0.4 g/kg; thiamine: 0.3 g/kg; pyridoxine: 0.3 g/kg; riboflavin: 0.2 g/kg), and other minor organic compounds (phytochelatins and enzymes). The two treatments sets, Commercial control and the Plant extract biostimulant, compared in this study are summarized in Table 3.

### 4.2. Harvest and Fruit Yield

Fruits were harvested by hand on 15 plants per plot when all of them reached a red-mahogany or darker skin color. Kordia was harvested on 23 December 2017, while Regina was harvested on 8 January 2018. Fruits were weighed to determine the yield per hectare considering planting density.

### 4.3. Fruit Fresh Weight, Size and Qualitative Traits

For each treatment, the fresh weight and the diameter of 200 randomly sampled fruits were individually measured with a digital scale and a digital Vernier caliper, respectively. Based on their diameters (expressed in mm), individual fruits were assigned to one of seven commercial size classes (<22, 22–24; 24–26; 26–28; 28–30; 30–32; >32 mm). For each treatment, the distribution of fruits into commercial size classes was used to estimate the number of fruits per tree (crop load). After these measurements, the fruits were divided in three subsamples (two of 50 fruits and one of 100 fruits). The first 50-fruit subsample was used for measuring individual fruit firmness with the Firmtech device (FT7, UP Umweltanalytische Produkte GmbH, Ibbenbüren, Germany), whereas the other 50 fruits were used to individually evaluate skin color by visual analysis and using a common export scale as a reference (ASOEX, Las Condes, Chile). This scale includes the following classes: light red, red, red mahogany, mahogany, dark mahogany.

The last 100 fruits were squeezed and tested individually for soluble solids content of flesh juice with a digital refractometer (MODEL HI96801, HANNA, Woonsocket, RI, USA).

Analysis of calcium in the fruits was performed by atomic absorption spectrometry, using flame atomic absorption spectroscopy. This system was equipped with a hollow monoelement cathode lamp (Hollow Cathode Lamp, Cambridge, UK) for analysis of calcium element [72].

### 4.4. Composition of Leaf Petiole Sap

An analysis of nutrient concentration in the petiole sap (Ca, K, Na, NH_4_, NO_3_ and Mg) was conducted using the Multi-ion 10 equipment (IMACIMUS, NT Sensors, El Catllar, SPAIN). One-hundred leaf petioles were randomly chosen for sampling in each plot on 16 November 2017 and washed with distilled water to remove dust particles and other residues that could affect the measurement. Then, they were pressed with a manual press to extract 1 mL of sap which was diluted in 19 mL of distilled water and analyzed with a sensor. This measurement was expressed in concentration of nutrients (mg/L).

### 4.5. Statistical Analysis

The significance of the differences between treatments in all the measured parameters was assessed with the t-student test, using IBM SPSS Statistics software (IBM Corp., Armonk, NY, USA).

## 5. Conclusions

The results of this study suggested, for the first time, that the foliar application of tropical plant extracts at very early phenological stages (between full bloom and fruit set) may be a suitable management practice to improve fruit yield components and fruit qualitative traits in commercial sweet cherry orchards, compared to the foliar application of a cytokinin promoter at the same phenological stages. However, the yield components (number of fruits per tree and fruit size) were differentially affected in Kordia and Regina trees. In addition, in Kordia this biostimulant-based product also induced a very interesting improvement in several qualitative traits that can positively affect the consumer acceptance of cherries (soluble solids content, fruit firmness), the fruit nutritional value (anthocyanin concentration), the post-harvest performances of the fruit (increasing fruit suitability for handling and transport), and the resistance to cracking. The Regina cultivar appears to be less responsive in this respect, but probably because of an indirect negative effect on these traits of the increased fruit set. In general, the results of our study suggest that the tested TPEB may be a sustainable and effective candidate alternative to the exogenous application of synthetic hormones for sweet cherry cultivation. However, additional research will be required to (a) better understand the mechanism underlying the complex cherry tree responses to this biostimulant, (b) fine-tune the application strategy of this plant extract by studying the dose and phenological stage of application, and (c) test the responses of other sweet cherry cultivars and fruit tree species to this promising class of biostimulants.

## Figures and Tables

**Table 1:** Cherry fruit yield components (mean ± standard error of the mean) in “Kordia” and “Regina” with two managing practices, Commercial control and Tropical-plant extract biostimulant (TPEB).

Treatment	Kordia		Regina	
	Fruit Yield (tons/ha)	Crop Load (Number Fruits/Tree)	Fruit Yield (tons/ha)	Crop Load (Number Fruits/Tree)
Commercial Control	12.064 ± 0.308	1113 ± 28	26.914 ± 0.449	3134 ± 54
TPEB	12.954 ± 0.267	1136 ± 23	30.438 ± 0.304	3369 ± 34
Significance	*	n.s.	**	**

Asterisks represent significant differences according to *t*-test, n.s. (not significant), * (0.05), ** (0.01).

**Table 2:** Fruit diameter, fruit fresh weight, soluble solids content (SSC), flesh firmness and calcium content (mean ± standard error of the mean) in “Kordia” and “Regina” with two managing practices, Commercial control and Tropical-plant extract biostimulant (TPEB).

Treatment	Kordia					Regina				
	Fruit Diameter (mm)	Fruit Fresh Weight (g/Fruit)	SSC (°Brix)	Flesh Firmness (g/mm^2^)	Calcium Content (mm/100 g)	Fruit Diameter (mm)	Fruit Fresh Weight (g/Fruit)	SSC (°Brix)	Flesh Firmness (g/mm^2^)	Calcium Content (mm/100 g)
Commercial Control	27.84 ± 0.26	12.08 ± 0.21	18.31 ± 0.28	325.29 ± 7.29	12.47 ± 0.19	25.42 ± 0.19	9.67 ± 0.19	18.86 ± 0.26	322.77 ± 6.69	12.02 ± 0.29
TPEB	28.55 ± 0.16	11.39 ± 0.19	20.47 ± 0.37	347.09 ± 7.66	15.75 ± 0.18	25.97 ± 0.19	10.46 ± 0.20	17.46 ± 0.22	324.45 ± 7.00	12.80 ± 0.18
Significance	*	*	***	*	*	*	**	***	n.s.	n.s.

Asterisks represent significant differences according to *t*-test, n.s. (not significant), * (0.05), ** (0.01), *** (0.001).

**Table 3:** Products sprayed at each of the four phenological stages in the Commercial control and the Tropical-plant extract biostimulant (TPEB) treatments.

Treatment	Phenological Stage			
	Early Stage D “Sepals Open”	Stage F “Full Bloom”	Stage G ”Full Petal Fall”	7 Days after Stage G
Commercial control	Seaweed extract (3.75 L/ha) + Organic fertilizer (14% of calcium complexed with 6% of amino acids; 4.5 L/ha)	Cytokinin promoter (2 L/ha) + Organic fertilizer (14% of calcium complexed with 6% of amino acids; 4.5 L/ha)	Cytokinin promoter (2 L/ha) + Organic fertilizer (14% of calcium complexed with 6% of amino acids; 4.5 L/ha)	Cytokinin promoter (2 L/ha) + Organic fertilizer (14% of calcium complexed with 6% of amino acids; 4.5 L/ha)
TPEB	Seaweed extract (3.75 L/ha) + Organic fertilizer (5% of calcium, CaO, and 5% of vegetal amino acids; 3 L/ha)	Tropical-plant extract (1.5 L/ha) + Organic fertilizer (5% of calcium, CaO, and 5% of vegetal amino acids; 3 L/ha)	Tropical-plant extract (1.5 L/ha) + Organic fertilizer (5% of calcium, CaO, and 5% of vegetal amino acids; 3 L/ha)	Tropical-plant extract (1.5 L/ha) + Organic fertilizer (5% of calcium, CaO, and 5% of vegetal amino acids; 3 L/ha)

## Data Availability

The datasets generated for this study are available on request to the corresponding author.
